# Electronic interactions between a stable electride and a nano-alloy control the chemoselective reduction reaction†
†Electronic supplementary information (ESI) available. See DOI: 10.1039/c6sc01864e


**DOI:** 10.1039/c6sc01864e

**Published:** 2016-05-24

**Authors:** Tian-Nan Ye, Jiang Li, Masaaki Kitano, Masato Sasase, Hideo Hosono

**Affiliations:** a Materials Research Center for Element Strategy , Tokyo Institute of Technology , 4259 Nagatsuta, Midori-ku , Yokohama 226-8503 , Japan . Email: kitano.m.aa@m.titech.ac.jp ; Email: hosono@msl.titech.ac.jp; b Laboratory for Materials and Structures , Tokyo Institute of Technology , 4259 Nagatsuta, Midori-ku , Yokohama 226-8503 , Japan; c ACCEL , Japan Science and Technology Agency , 4-1-8 Honcho, Kawaguchi , Saitama 332-0012 , Japan

## Abstract

Controlling the electronic structure of heterogeneous metal catalysts is considered an efficient method to optimize catalytic activity. Here, we introduce a new electronic effect induced by the synergy of a stable electride and bimetallic nanoparticles for a chemoselective reduction reaction. The electride [Ca_24_Al_28_O_64_]^4+^·(e^–^)_4_, with extremely low work function, promotes the superior activity and selectivity of a Ru–Fe nano-alloy for the conversion of α,β-unsaturated aldehydes to unsaturated alcohols in a solvent-free system. The catalyst is easily separable from the product solution and reusable without notable deactivation. Mechanistic studies demonstrate that electron injection from the electride to the Ru–Fe bimetallic nanoparticles promotes H_2_ dissociation on the highly charged active metal and preferential adsorption of C=O bonds over C=Cs bond of the unsaturated aldehydes, to obtain the thermodynamically unfavorable but industrially important product.

## Abstract 2

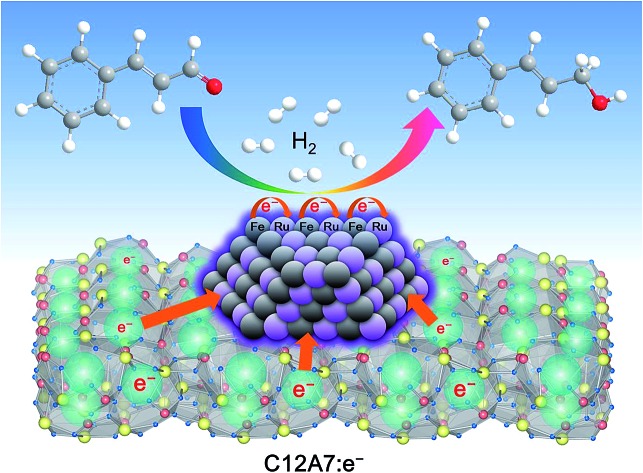
The electronic effects induced by the synergy of stable C12A7:e^–^ electride and bimetallic Ru–Fe nanoparticles efficiently control the chemoselective reduction reaction.

## Introduction

Numerous efforts have been devoted to achieving a high yield of required chemicals without any byproduct using a heterogeneous catalyst.^1^ Supported-metal catalysts have been studied extensively in heterogeneous catalysis.^2^ Metal–support interactions often lead to electronic and steric effects that determine the overall activity and selectivity of the catalyst.^3^ Thus, tuning the electronic structure of heterogeneous metal catalysts is considered an efficient strategy to improve catalytic performance. To achieve this, there have been many attempts to engineer the local electronic structure of heterogeneous catalysts using external modifiers such as alkali compounds and surface organic ligands.^4^ However, these compounds are inseparable from the liquid product, thermally unstable, or environmentally harmful.^5^ If a stable promoter that can precisely control the electronic properties of a metal catalyst could be found, then the performance of the catalyst would be increased significantly.

Electrides are ionic crystals with cavity-trapped electrons that act as anions. [Ca_24_Al_28_O_64_]^4+^·(e^–^)_4_ (C12A7:e^–^) is an inorganic electride with anionic electrons in the positively charged framework ([Ca_24_Al_28_O_64_]^4+^) that was created in 2003.^6^ The most characteristic feature of the C12A7:e^–^ electride is its low work function (2.4 eV), comparable to that of alkali metals, but with higher chemical inertness, which makes this material a promising electron donor in chemical reactions.^7^ We have reported that C12A7:e^–^ alone, or in combination with Ru, functions as an effective catalyst in chemical reactions, such as ammonia synthesis and decomposition, and CO_2_ splitting.^8^ However, there is no report of the application of C12A7:e^–^ as a catalyst for liquid phase reactions because they are chemically unstable in aqueous media and tend to release electrons into the solvent or moist environment. The C12A7:e^–^ electride has recently been used as an electron generator in aqueous solution to facilitate the pinacol coupling reaction of aldehydes, and the chemoselective reduction and oxidation of ketones.^9^ However, the electride acts as a kind of reducing agent in such reactions, and can thus only be used once due to consumption of the trapped electrons. Therefore, it is of interest to investigate the possibility of applying an electride-based catalyst to some important liquid phase organic reactions.

Chemoselective hydrogenation of α,β-unsaturated aldehydes to the corresponding unsaturated alcohols is difficult but fundamentally important in both chemical research and industry.^10^ The resulting alcohol is a versatile intermediate for the production of fine chemicals such as pharmaceuticals and fragrances.^11^ However, it is difficult to achieve high selectivity and activity for the unsaturated alcohol product because hydrogenation of the C=C bond over the C=O bond is both thermodynamically and kinetically favored.^12^ Conventional hydrogenation catalysts based on supported Pt, Pd, and Ru produce mainly saturated aldehydes.^13^ The chemoselectivity and activity for the reduction is strongly influenced by the catalyst properties, such as the metal particle size, electronic properties, metal–support interaction, and the addition of promoters.^14^ In addition, solvent effects are well documented in this type of reaction, *e.g.*, the adsorption of a polar reactant is enhanced by a non-polar solvent, and *vice versa*.^15^ However, there is a high demand for solvent-free reactions to realize high efficiency and environmentally benign processes.^16^

In this work we demonstrate that C12A7:e^–^ electride-supported Ru–Fe alloy nanoparticles (Ru–Fe/C12A7:e^–^) can act as a highly efficient and selective heterogeneous catalyst for the liquid phase chemoselective hydrogenation of α,β-unsaturated aldehydes under solvent-free conditions. After reaction completion, the solid Ru–Fe/C12A7:e^–^ can be easily separated from the reaction mixture and reused without decrease in catalytic efficiency. Detailed characterization indicates that the electronic effect induced by the electron donation of the C12A7:e^–^ electride support and the charge transfer between nano-alloy particles is responsible for the superior catalytic performance for chemoselective hydrogenation. To the best of our knowledge, this is the first report on an electride-based heterogeneous catalyst in a liquid catalytic reaction with a solvent-free system.

## Results and discussion

The Ru–Fe/C12A7:e^–^ catalysts were fabricated initially by conventional solid-phase reaction and subsequent chemical vapor deposition (CVD) of metal carbonyl complexes in a vacuum (Fig. S1†). The surface area of the prepared Ru–Fe/C12A7:e^–^ was only 0.8 m^2^ g^–1^, and the weight percentage of Ru–Fe deposited onto the C12A7:e^–^ electride was 1 wt% for each metal. Powder X-ray diffraction (XRD) patterns (Fig. S2†) of Ru–Fe/C12A7:e^–^ show no clear diffraction peaks due to Ru or Fe, which indicates the formation of small Ru–Fe nanoparticles. In order to confirm the formation of the Ru–Fe alloy, excess amounts of Ru and Fe were loaded on amorphous SiO_2_ by the same procedure as Ru–Fe/C12A7:e^–^. In the XRD pattern of 5 wt% Ru–5 wt% Fe/SiO_2_ (Fig. S3†), the observed peaks were identified as Ru–Fe alloy with a hexagonal close-packed (hcp) phase (space group: *P*6_3_/*mmc*).^17^ Therefore, it can be considered that hexagonal Ru–Fe alloy nanoparticles are formed on the surface of C12A7:e^–^. X-ray photoelectron spectroscopy (XPS; Fig. S4a and b†) of the samples at the Ru 3p and Fe 2p levels unambiguously revealed the formation of metallic Ru (461.5 eV) and Fe (707.1 eV), although a fraction of the Fe was oxidized (710.8 eV).^18^ The surface of C12A7:e^–^ was densely and uniformly covered by 15 nm diameter Ru–Fe nanoparticles, as confirmed by scanning electron microscopy (SEM; Fig. S5a and b†). The high density and homogeneous distribution of Ru–Fe nanoparticles may benefit their potential application as catalysts in heterogeneous systems.

The Ru–Fe/C12A7:e^–^ catalyst was firstly investigated using cinnamaldehyde as a model substrate. Ru/C12A7:e^–^ showed poor activity and Fe/C12A7:e^–^ gave zero conversion (Table 1, entries 1, 2). In contrast, the bimetallic Ru–Fe catalyst had substantially higher catalytic activity under the same conditions, giving 96.2% conversion of cinnamaldehyde and 96.7% cinnamyl alcohol selectivity, with the carbon balance >98.5% (entry 4). This demonstrates the synergistic effect of Ru–Fe bimetallic nanoparticles in contrast to the monometallic species; a physical mixture of the monometallic Ru and Fe catalysts showed little enhancement (entry 3). Note that the Ru–Fe/C12A7:e^–^ catalyst with a much lower specific surface area showed significantly higher catalytic activity and selectivity than Ru-based, Pt-based, and other transition metal based catalysts reported to date (Table S1†). Such a high catalytic performance is attributed to the combination of Ru–Fe alloy nanoparticles and C12A7:e^–^ with high electron donation ability. The reaction conditions were further optimized to 130 °C, 2.0 MPa H_2_, and 12 h to achieve optimal hydrogenation activity (Table S2,† entries 1–5).

**Table 1:** Catalytic performance for the chemoselective hydrogenation of cinnamaldehyde^*a*^

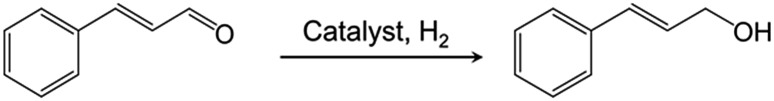			
Entry	Catalyst	Conversion [%]	Selectivity [%]
1^*b*^	Ru/C12A7:e^–^	32.9	82.2
2^*c*^	Fe/C12A7:e^–^	—	—
3	Ru/C12A7:e^–^ + Fe/C12A7:e^–^	48.8	89.9
4	Ru–Fe/C12A7:e^–^	96.2	96.7
5^*b*^	Ru/C12A7:O^2–^	28.1	52.6
6	Ru–Fe/C12A7:O^2–^	91.9	72.3
7	Ru–Fe/Al_2_O_3_	84.3	74.3
8	C12A7:e^–^	—	—
9	Blank	—	—

^*a*^Typical conditions: 8 mmol substrate, 100 mg catalyst (1 wt% Ru, 1 wt% Fe), H_2_ (2.0 MPa), 130 °C, 12 h, *N*_e_ = 2.2 × 10^21^ cm^–3^.

^*b*^2 wt% Ru.

^*c*^2 wt% Fe.

The product cinnamyl alcohol when used as a substrate gave no conversion, which also confirms the high selectivity of Ru–Fe/C12A7:e^–^ for the desired transformation (Table S2,† entry 6). The size effect (Fig. S6†) and weight ratio (Fig. S7†) of the Ru–Fe alloy nanoparticles also affect the performance of the catalyst. The 1 wt% Ru–1 wt% Fe/C12A7:e^–^ catalyst was found to exhibit the highest selectivity and activity for this reaction.

**Fig. 1:**
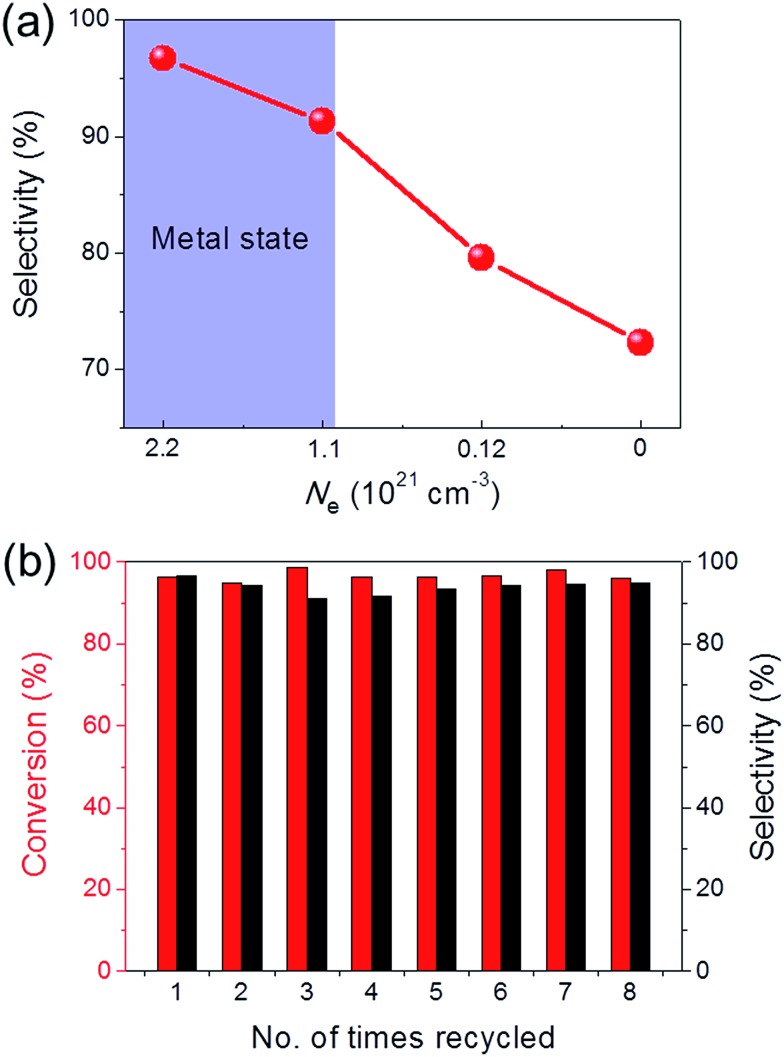
(a) Cinnamaldehyde hydrogenation selectivity with Ru–Fe loaded C12A7:e^–^/O^2–^ catalysts as a function of electron concentration. (b) Recycling experiment for the selective hydrogenation of cinnamaldehyde over Ru–Fe/C12A7:e^–^. Reaction conditions: 8 mmol substrate, 100 mg catalyst, H_2_ (2.0 MPa), 130 °C, 12 h.

Ru/C12A7:O^2–^ (with an O^2–^ ion in the cage in place of two electrons) and Ru/C12A7:e^–^ exhibited poor activity and selectivity for cinnamyl alcohol (Table 1, entries 1, 5), due to the intrinsically low activity and selectivity of Ru catalysts for this reaction. Although the catalytic activity is greatly enhanced when Fe is co-loaded with Ru on C12A7:O^2–^, the selectivity is still much lower than that of Ru–Fe/C12A7:e^–^ (Table 1, entry 6). Interestingly, the high selectivity of Ru–Fe/C12A7:e^–^ provides information on the critical electron concentration (*N*_e_) of C12A7:e^–^ as a support (Fig. S8 and 9, Table S3†). *N*_e_ > 1.1 × 10^21^ cm^–3^ significantly increases the selectivity compared with *N*_e_ = 1.2 × 10^20^ cm^–3^ (Fig. 1a). Note that a metal–insulator transition occurs in bulk C12A7:e^–^ at around 1 × 10^21^ cm^–3^.^19^ This observation suggests that the selectivity of this reaction is directly proportional to *N*_e_, which is absolutely related to the electron donation ability of the support. This trend was also observed for the promoted activity of Ru-loaded C12A7:e^–^ for ammonia synthesis.8*c* Ru–Fe loaded Al_2_O_3_ also showed relatively poor performance for this reaction (Table 1, entry 7). All of these results suggest that selectivity for the hydrogenation of α,β-unsaturated aldehydes is substantially improved by the electronic promoting effect of the C12A7:e^–^ support with high electron density. Bare C12A7:e^–^ without metal catalyst loading gave no conversion under the same conditions (Table 1, entries 8, 9), which indicates the key importance of the metal–support hetero-nanostructures to drive the hydrogenation reaction. It is well known that the selectivity for hydrogenation of unsaturated aldehydes is highly improved in the presence of external modifiers such as alkali metal compounds (KOH and NaOH) and surface organic ligands (phosphines, arsines, and amines).^20^ In our case, high selectivity was achieved by the combination of Ru–Fe alloy nanoparticles and C12A7:e^–^ without such inseparable additives.

The sustainability of catalysts is one of the most important properties for practical applications. To test the sustainability of Ru–Fe/C12A7:e^–^ in the catalytic reaction, the used catalyst was separated from the reaction solution *via* filtration or centrifugation. Ru–Fe/C12A7:e^–^ was successfully reused for more than eight cycles without any obvious loss of catalytic activity for the hydrogenation of cinnamaldehyde (Fig. 1b); the conversion was 96.1% in the eighth cycle, with 94.9% selectivity for cinnamyl alcohol. These results show the excellent sustainability of the Ru–Fe/C12A7:e^–^ catalyst.

In a further set of experiments, we focused on the selective hydrogenation of other α,β-unsaturated aldehydes (Table 2). Ru–Fe/C12A7:e^–^ could promote the hydrogenation of a series of substrates with moderate to high conversion and excellent selectivity towards the corresponding unsaturated alcohols. α-Methyl cinnamaldehyde was effectively converted to the corresponding unsaturated alcohol with a high selectivity of over 95.2% (Table 2, entry 1). Although the hydrogenation of unsaturated aldehydes with methoxy and halo groups is more difficult than that of cinnamaldehyde, excellent conversion and selectivity for the corresponding unsaturated alcohols could be achieved after prolonged reaction times (entries 2–5). Due to the high melting point of 4-nitrocinnamaldehyde, the hydrogenation reactions were performed under the same conditions as for cinnamaldehyde but in THF solvent. Notably, the chemoselective hydrogenation of 4-nitrocinnamaldehyde also proceeded without difficulty (entry 6). Ru–Fe/C12A7:e^–^ could also be applied to the hydrogenation of aliphatic α,β-unsaturated aldehydes such as citral, *trans*-2-heptenal, *trans*-2-hexenal, crotonaldehyde and 3-methyl-2-butenal (entries 7–11). As expected, all substrates were quantitatively reduced to their corresponding alcohols under optimized catalytic conditions. The reaction was sensitive to steric hindrance of the substituents on the alpha sites, and as a result *trans*-2-methyl-2-pentenal and *trans*-2-methyl-2-butenal exhibited relatively low conversion under relatively rigorous conditions (entries 12, 13), but still with high selectivity. In contrast, the activity of the α,β-unsaturated ketone hydrogenation reaction was quite poor due to steric hindrance from the methyl group in the carbonyl component (Table S4,† entry 1). Nevertheless, these results demonstrate that the electride supported Ru–Fe catalyst exhibits high chemoselectivity for the hydrogenation of various α,β-unsaturated aldehydes in a solvent-free system.

**Table 2:** Chemoselective hydrogenation of various α,β-unsaturated aldehydes using Ru–Fe/C12A7:e^–^^*a*^

Entry	Substrate	Product	Conversion [%]	Selectivity [%]
1	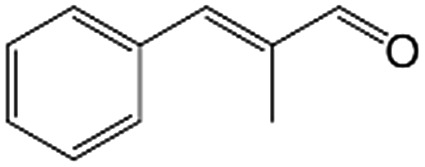	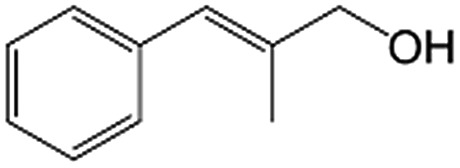	88.9	95.2
2^*b*^	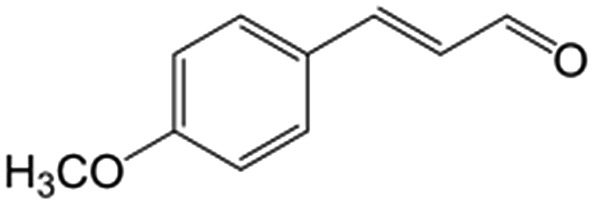	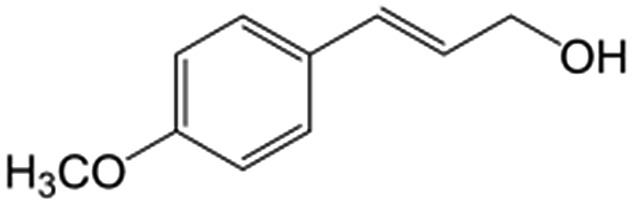	89.2	93.6
3^*b*^	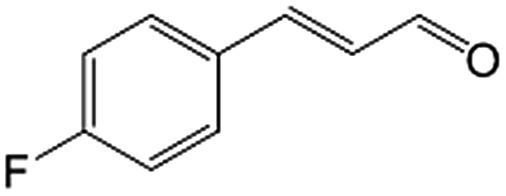	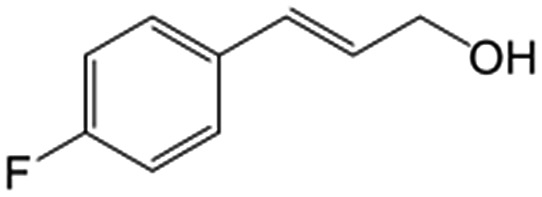	92.2	95.4
4^*b*^	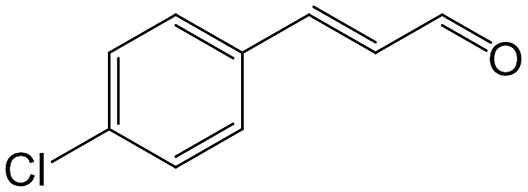	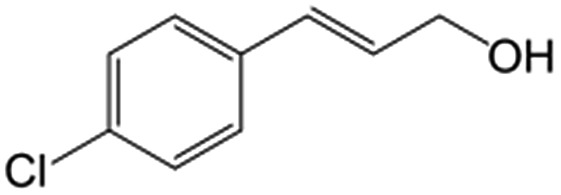	91.3	94.2
5^*b*^	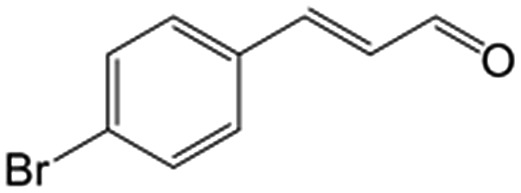	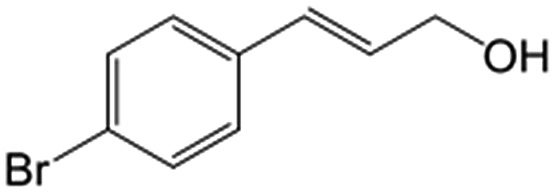	85.5	91.4
6^*c*^	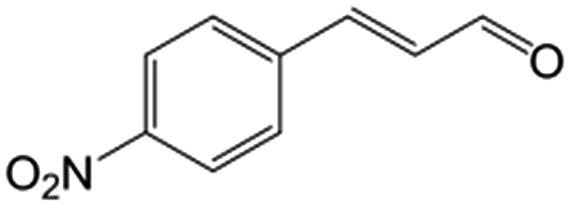	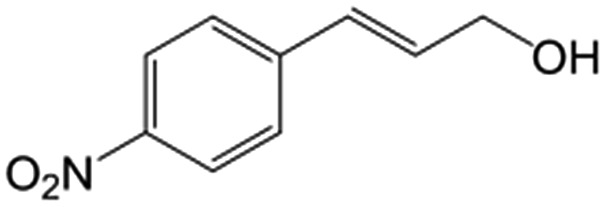	73.1	91.7
7^*d*^	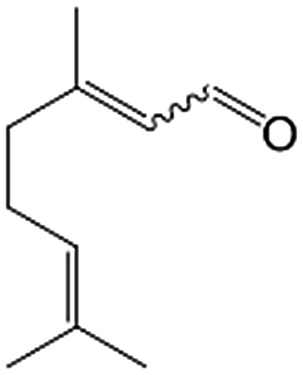	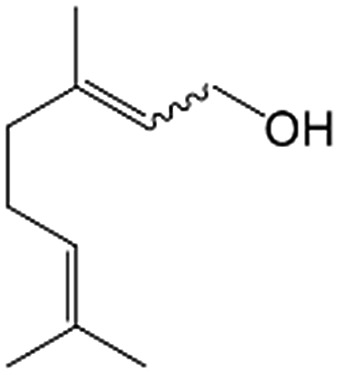	83	92.9
8^*e*^	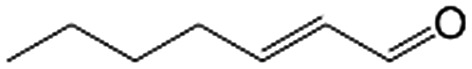	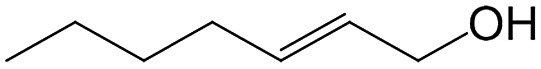	93.4	96.8
9^*e*^	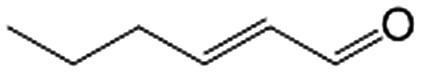	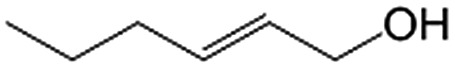	97.8	95.5
10^*f*^	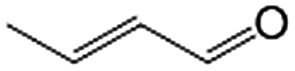	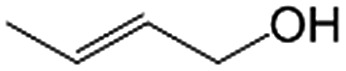	90.1	75.0
11^*e*^	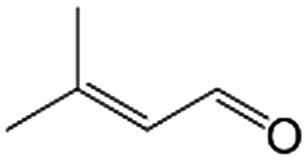	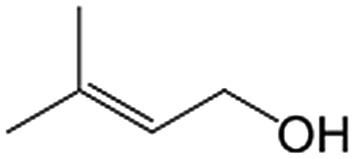	96.7	93.1
12^*f*^	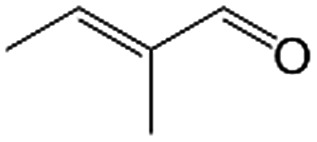	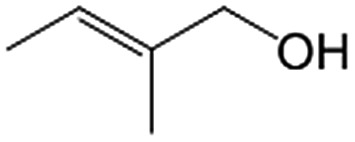	44.1	87.3
13^*g*^	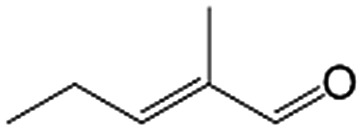	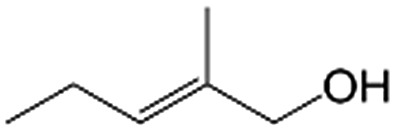	39.6	96.4

^*a*^Typical conditions: 8 mmol substrate, 100 mg catalyst, H_2_ (2.0 MPa), 130 °C, 12 h.

^*b*^48 h.

^*c*^4 mmol substrate, 5 ml THF, 48 h.

^*d*^110 °C, 12 h.

^*e*^4 mmol substrate, 90 °C, 36 h.

^*f*^H_2_ (4.0 MPa), 48 h.

^*g*^36 h.

**Fig. 2:**
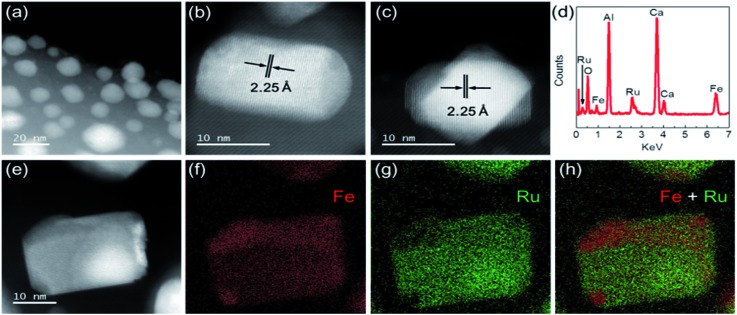
HAADF-STEM images of Ru–Fe/C12A7:e^–^. (a) Overview image of Ru–Fe/C12A7:e^–^. (b, c) Representative images and (d) STEM-EDX spectrum of Ru–Fe nanoparticles on the surface of C12A7:e^–^. (e) STEM image of Ru–Fe/C12A7:e^–^ and EDX elemental maps for (f) Fe, (g) Ru, and (h) Fe + Ru.

To elucidate the superior performance of Ru–Fe/C12A7:e^–^ for the chemoselective hydrogenation of α,β-unsaturated aldehydes, the detailed structural and electronic features of the catalysts were analyzed. The composition and nanostructure of Ru–Fe/C12A7:e^–^ was studied using aberration-corrected (AC) high-angle annular dark field scanning transmission electron microscopy (HAADF-STEM). Fig. 2a shows Ru–Fe nanoparticles with a mean size of 15 nm formed on the surface of the C12A7:e^–^ electride, which is consistent with scanning electron microscopy (SEM) analysis (Fig. S5a and b†). The plane spacing was estimated from detailed observations (Fig. 2b and c) to be 2.25 Å, close to 2.28 Å of RuFe (100) with hcp structure (space group: *P*6_3_/*mmc*), which is consistent with the results of XRD measurement (Fig. S3†). No separate Ru or Fe lattices were detected, which indicates that Ru and Fe are distributed homogeneously in the Ru–Fe nanoparticles. Energy dispersive X-ray spectroscopy (EDX) analysis (Fig. 2d–h) revealed that the Ru signal intensity relative to Fe was almost the same at different points in the nanoparticles, *i.e.*, an atomic-level Ru–Fe alloy is formed.

**Fig. 3:**
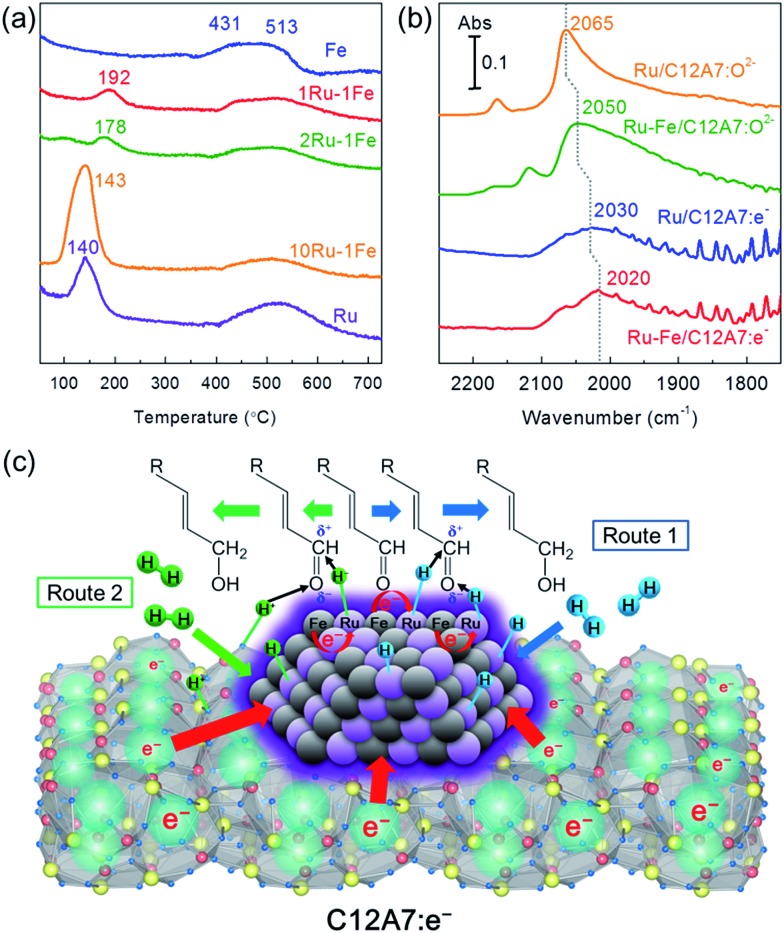
(a) H_2_-TPR profiles for several bimetallic Ru–Fe/C12A7:e^–^ catalysts with various Ru/Fe weight ratios: 2 wt% Fe/C12A7:e^–^, 1 wt% Ru–1 wt% Fe/C12A7:e^–^, 2 wt% Ru–1 wt% Fe/C12A7:e^–^, 10 wt% Ru–1 wt% Fe/C12A7:e^–^, and 2 wt% Ru/C12A7:e^–^. (b) Difference DRIFTS spectra for adsorption of CO onto Ru/C12A7:O^2–^, Ru–Fe/C12A7:O^2–^, Ru/C12A7:e^–^, and Ru–Fe/C12A7:e^–^ at –170 °C under 5 kPa of CO. (c) Possible pathway for chemoselective hydrogenation of α,β-unsaturated aldehydes over Ru–Fe/C12A7:e^–^.

The reducibility of the catalysts was investigated at various metal weight ratios using hydrogen temperature-programmed reduction (H_2_-TPR) to understand the interaction between Ru and Fe in the alloy. As shown in Fig. 3a, the mono-Ru catalyst gives a single peak at *ca.* 140 °C, which is assigned to the reduction of RuO_2_.^21^ For the mono-Fe sample, two distinct peaks around 430 and 515 °C were attributed to the reduction of FeO_*x*_ species.^22^ For a series of Ru–Fe bimetallic catalysts, one hydrogen consumption peak was observed in each sample, which shifted from 143 to 192 °C with a decrease in the Ru/Fe weight ratio, indicating a significant synergistic effect between Ru and Fe. The reduction temperature of oxidizable metals such as Fe is lowered by the presence of noble metals in close proximity.12a,^23^ In addition, each sample had a broadened hydrogen consumption peak in the temperature range of 427–677 °C, which was ascribed to the incorporation of H_2_ into C12A7:e^–^ cages as H^–^ ions during the high temperature stage.8*b*

The electron donation capabilities of Ru–Fe nanoparticles on the C12A7:e^–^ electride was examined by diffuse reflectance infrared Fourier transform (DRIFT) spectroscopy using CO as a probe molecule. As shown in Fig. 3b, Ru/C12A7:O^2–^ exhibits main peaks at around 2100–2000 cm^–1^, which can be assigned to the C–O stretching vibration of linearly adsorbed CO on Ru^0^ sites, indicating that there is no electron transfer from C12A7:O^2–^ to the Ru metal nanoparticles. This is due to the fact that C12A7:O^2–^ is a typical insulator material, in which O^2–^ ions are incorporated as counter anions to the positively charged [Ca_24_Al_28_O_64_]^4+^ lattice framework composed of subnanometer-sized cages.^24^ The high frequency band centered at 2165 cm^–1^ corresponds to tricarbonyl species on partially oxidized Ru sites.^25^ Fe/C12A7:O^2–^ has no adsorption peaks, which indicates that the interaction between CO molecules and Fe is quite weak (Fig. S10†). Notably, the linear Ru^0^–CO peak was shifted to a slightly lower frequency (2050 cm^–1^) after Fe addition. The red-shift of the CO signal is clear evidence of the electronic interaction between Ru and Fe species. Electropositive Fe metal generally acts as an electron-donating ligand that increases the electron density of Ru, thereby favoring the back-donation of electrons to the 2π* antibonding orbitals of CO, which accounts for the C≡O bond weakening (red-shift of the CO stretching band). In contrast, the electron density of Fe atoms decreases due to the electron transfer from Fe to Ru. A similar electron transfer effect is demonstrated on a model surface of Pt_80_Fe_20_(111) by Hückel calculations.^26^ Most importantly, the electronic effect of the support also contributes to the C≡O bond weakening on both Ru/C12A7:e^–^ and Ru–Fe/C12A7:e^–^. Compared to Ru/C12A7:O^2–^ and Ru–Fe/C12A7:O^2–^, a clear red-shift is observed for the linear Ru^0^–CO peak (2030 cm^–1^) of Ru/C12A7:e^–^ and for that (2020 cm^–1^) of Ru–Fe/C12A7:e^–^, respectively (Fig. 3b), which implies the C≡O bond of a CO molecule adsorbed on Ru/C12A7:e^–^ and Ru–Fe/C12A7:e^–^ is weakened by the electrons encaged in C12A7:e^–^, which has an extremely low work function and metallic conductivity.^6^ These results provide direct evidence for the strong electronic modification of the active metal by the C12A7:e^–^ electride.

The proposed reaction mechanism for the chemoselective hydrogenation of α,β-unsaturated aldehydes over Ru–Fe/C12A7:e^–^ is shown in Fig. 3c. It is well known that the adsorption geometry of the unsaturated aldehyde on the catalyst surface is very important in determining the selectivity for the unsaturated alcohol.^27^ Both C=C and C=O bonds can interact with the metal surface and the adsorption mode is strongly dependent on the surface of the metal catalyst. In our case, the electronic nature of the metal nanoparticles can be modified by the electron donation from C12A7:e^–^, leading to the marked increase in the hydrogenation selectivity. The electron enrichment of the metal surface decreases the binding energy of the C=C bond *via* increased repulsive interaction between metal d-orbitals and the C=C bond, favoring a vertical adsorption configuration *via* the C=O bond (Fig. 3c).^28^ This repulsive interaction and adsorption configuration is theoretically and experimentally demonstrated for various catalysts.^26^,27a,^29^ To understand the interaction between catalysts and the reactant molecules, the reaction order with respect to the initial reactant concentration was investigated (Fig. S11†). Zero order dependence on the initial cinnamaldehyde concentration was observed for Ru–Fe/C12A7:e^–^, Ru–Fe/C12A7:O^2–^, and Ru/C12A7:e^–^, indicating that cinnamaldehyde molecules adsorb strongly on the surface of these catalysts.^30^ These results suggest that the repulsive interaction has no influence on the aldehyde adsorption ability of the catalyst surface and changes only the adsorption geometry of the substrate, *i.e.*, the cinnamaldehyde is preferentially adsorbed on metal particles through the C=O bond. Additionally, electropositive Fe sites are formed in the Ru–Fe bimetallic system as demonstrated in FT-IR analysis (Fig. 3b). The Fe sites act as electrophilic sites and activate the C=O bond *via* the lone electron pair of the oxygen atom, which leads to a weakening of the C=O bond.20c,27a,28a The hydrogenation of C=O bonds is thus enhanced compared with the C=C bonds. There are two possible routes to cleave H_2_. H_2_ dissociation occurs preferentially on the Ru surface due to the low dissociation barrier of H_2_ on Ru, which results in the formation of nonpolar hydrogen species *via* homolytic cleavage of H_2_ (Fig. 3c, Route 1). Typically, H adatoms (nonpolar hydrogen species) readily react with both C=C and C=O bonds, with the hydrogenation of C=C bond being thermodynamically favorable. However, the hydrogenation of C=O bond selectively occurs over Ru–Fe/C12A7:e^–^ because the unsaturated aldehyde molecules are adsorbed on the catalyst surface *via* the vertical configuration.

Another possibility is heterolytic H_2_ dissociation (H_2_ → H^–^ + H^+^) at the interface between a metal and basic support, which would subsequently enhance selective hydrogenation reactions.^31^ Selective hydrogenation can be achieved using stoichiometric amounts of metal hydrides such as NaBH_4_ and LiAlH_4_*via* nucleophilic attack by H^–^ species to the positively charged carbon of the C=O bond.^32^ In the case of Ru–Fe/C12A7:e^–^, it can be expected that H^–^ species are formed on electron-rich metal sites and H^+^ is simultaneously formed on a framework oxygen at the surface of C12A7:e^–^. The resulting H^–^ and H^+^ species react with the carbon and oxygen sites of C^*δ*+^=O^*δ*–^ bonds, respectively (Fig. 3c, Route 2). The electride-loaded alloy catalysts would then lead to complete chemoselective reduction of polar functionalities while retaining the C=C bonds. Although the detailed mechanism remains to be clarified at this stage, it is important to emphasize that electron transfer between electride and metal nanoparticles could be the key factor in the chemoselective reduction of unsaturated aldehydes.

## Conclusions

In summary, Ru–Fe alloy nanoparticles deposited on C12A7:e^–^ electride were constructed to achieve highly efficient solvent-free hydrogenation of α,β-unsaturated aldehydes. The intrinsically low work function C12A7:e^–^ injects electrons into the active Ru–Fe nanoparticles, which leads to the formation of the unsaturated alcohol with outstanding catalytic activity and high selectivity, without the need for basic additives in the reaction solution. The synergistic effect of the alloy metal Ru and Fe offers atom-scale electron transfer to activate Ru and induces electrophilic activation towards C=O, both favoring chemoselective hydrogenation. The electride-based catalyst also exhibited excellent sustainability and superior chemoselectivity of over 95% during long-term cycling. These results could serve as inspiration for the further exploitation of electride-based metal or alloy catalyst interactions in the design and synthesis of novel heterogeneous catalysts. We consider that a family of electrides with high electron donation ability could find wide application in different fields of catalysis.

## Supplementary Material

Supplementary information
