# Supplementary material for: Electronic interactions between a stable electride and a nano-alloy control the chemoselective reduction reaction
Source: Chem Sci. 2016 May 24;7(9):5969–75. doi: 10.1039/c6sc01864e (PMC6022174; doi:10.1039/c6sc01864e)
Supplement: Supplementary file 1 [file SC-007-C6SC01864E-s001.pdf]

Electronic Supplementary Material (ESI) for Chemical Science.
This journal is © The Royal Society of Chemistry 2016

Electronic Interactions between Stable Electride and Nano-alloy Control the Chemoselective Reduction Reaction

Tian-Nan Ye, Jiang Li, Masaaki Kitano, Masato Sasase, Hideo Hosono**

Materials Research Center for Element Strategy, Tokyo Institute of Technology, 4259 Nagatsuta, Midori-ku, Yokohama 226-8503, Japan

Frontier Research Center, Tokyo Institute of Technology, 4259 Nagatsuta, Midori-ku, Yokohama 226-8503, Japan

ACCEL, Japan Science and Technology Agency, 4-1-8 Honcho, Kawaguchi, Saitama, 332-0012, Japan

Methods

Preparation of Ru-Fe/C12A7:e$^-$

C12A7:e$^-$ electride powders were prepared by the reaction of C12A7, CaO·Al_2O_3 (CA) and Ca metal at 1100 °C. Ru and Fe loading was conducted by chemical vapor deposition (CVD) process using their corresponding metal carbonyl $Ru_3(CO)_{12}$ and $Fe_2(CO)_9$ precursors. In a typical catalyst synthesis, C12A7:e$^-$ electride powders with $Ru_3(CO)_{12}$ and $Fe_2(CO)_9$ were sealed in an evacuated silica tube and heated under a temperature program as shown in Fig. S1.

Hydrogenation of α,β-unsaturated aldehydes

All reactions were carried out in a 25-mL stainless steel autoclave fitted with a glass mantel, 60-bar manometer, and a magnetic stirrer. In a typical reaction, 100 mg of catalyst and 8 mmol substrates were sealed in the autoclave. Then autoclave was flushed three times with H_2, pressurized with H_2 (20 bar), and heated to 130 °C over 30 min with stirring (800 rpm). The reactor was held at this temperature for 12 h. The products were analyzed by gas chromatography (GC) with an external standard of benzyl alcohol and the identity of the products was further confirmed by GC-MS.

Characterization.

Samples for examination by scanning transmission electron microscopy (STEM) were prepared by dispersing the dry catalyst powder onto a holey carbon film supported by a 300 mesh copper TEM grid. STEM high angle annular dark field (HAADF) images of the supported metal nanoparticles were obtained using an aberration corrected JEM ARM-200 F STEM operating at 200 kV. X-ray energy dispersive (XEDS) spectra were acquired from individual metal nanoparticles 41 nm in size by rastering the beam over the entire metal particle, while using a JEOL Centurio 0.9sr silicon drift detector. The sample powders were also dispersed onto an Al-stub and examined in SE and backscatter mode in a JEOL JSM-7600F scanning electron microscope (SEM) equipped with an EDAX energy dispersive X-ray spectrometer. Transmission electron microscopy (TEM) measurements were conducted in bright field imaging mode using a Tecnai 20FEG transmission electron microscope operating at 200 kV. The mean Ru particle diameters were calculated by determining the size of more than 200 particles per sample using iTEM software (soft Imaging System GmbH).

X-ray photoelectron spectroscopy (XPS, ESCA-3200, Shimadzu) measurements were carried out using Mg Ka radiation at < 10^{-6} Pa (applied bias voltage to X-ray source of 8 kV).

The Brunauer–Emmett–Teller specific surface areas of the samples were determined by the measurement of nitrogen adsorption–desorption isotherms at −196 °C using an automatic gas-adsorption instrument (BELSORP-mini II, MiccrotracBEL) after evacuation of the samples at 150 °C. Ru content was determined by inductively coupled plasma atomic emission spectroscopy (ICP-AES; ICPS-8100, Shimadzu).

H_2-temperature-programmed reduction (H_2-TPR) profiles were measured in a BELCAT-A instrument (MiccrotracBEL, Japan). A 500 mg samples were pretreated in O_2 (30 mL/min) at 623 K for 30 min to remove CO adsorbed on the surface and then cooled to room temperature. Subsequently, reducing gas composed of 5% H_2/95% Ar was employed at a flow rate of 30 mL/min and a heating rate of 10 K/min from ambient to 1173 K. The consumption of H_2 was monitored by a thermal conductivity detector (TCD) and mass spectrometer (Bell Mass, MiccrotracBEL, Japan). To investigate the oxidation and reduction of the bimetallic system during the H_2-TPR characterization, we investigated the structure of 5wt%Ru-5wt%Fe/SiO_2 by XRD analysis after the pretreatment the same as Ru-Fe/C12A7:e− and re-reduction in 5% H_2/95% Ar, respectively (Fig. S12).

FT-IR spectra of adsorbed CO were measured using a spectrometer (FT/IR-6100, Jasco) equipped with a mercury–cadmium–tellurium detector at a resolution of 4 cm^{-1}. Samples were pressed into self-supported disks. A disk was placed in a silica-glass cell equipped with KBr windows and connected to a closed gas-circulation system to allow thermal adsorption–desorption experiments. The disk was pretreated with circulated H_2 at 350 °C for 2 hours and then cooled to room temperature. After the pretreatment, the disk was cooled to −170°C under vacuum to obtain a background spectrum. Pure CO (99.99999%) were supplied to the system through a liquid-nitrogen trap. The infrared spectrum of the sample at −170°C prior to CO adsorption was used as the background for difference spectra obtained by subtracting the backgrounds from the spectra of CO-adsorbed samples.

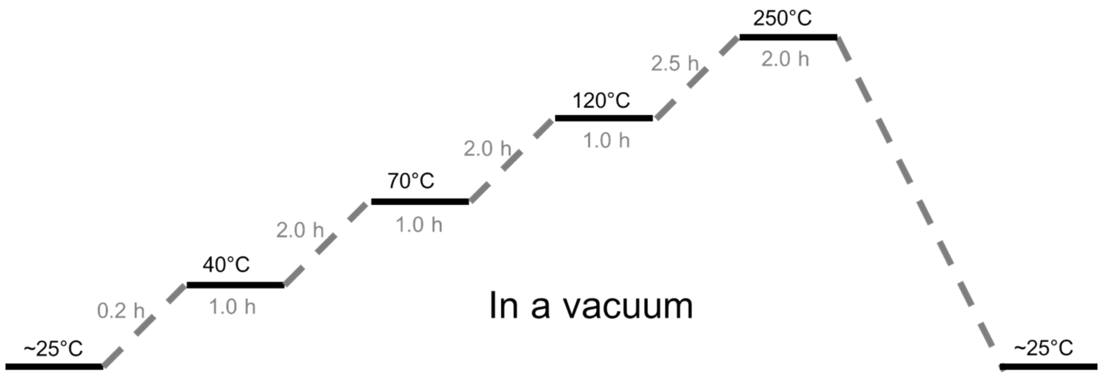

Fig. S1 Detailed synthetic parameters for preparing Ru-Fe/C12A7:e⁻.

Heated temperature program: 2 °C min^{-1} up to 40 °C, held for one hour, 0.25 °C min^{-1} up to 70 °C, held for one hour, 0.4 °C min^{-1} up to 120 °C, held for one hour, 0.9 °C min^{-1} up to 250 °C, held for two hours and then cooled to ambient temperature. The all program was operated under vacuum.

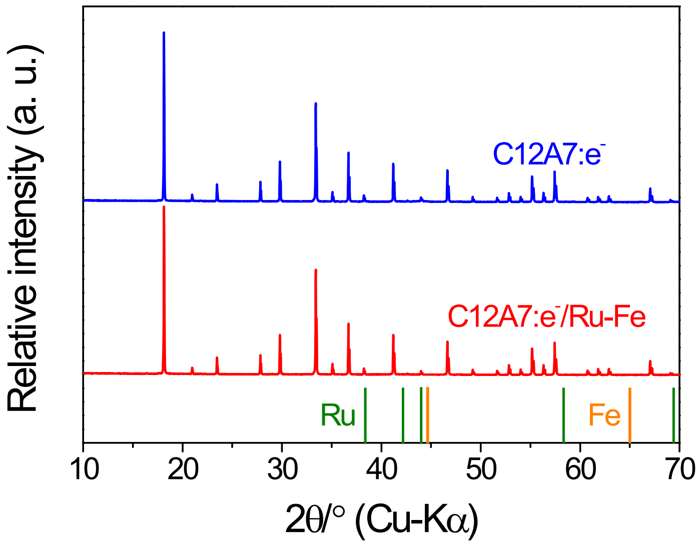

Fig. S2 XRD patterns of pure C12A7:e⁻ and Ru-Fe/C12A7:e⁻. The bottom green columns and orange columns represent the reference XRD patterns of Fe (JCPDS No.65-4899) and Ru (JCPDS No.65-1863) metal.

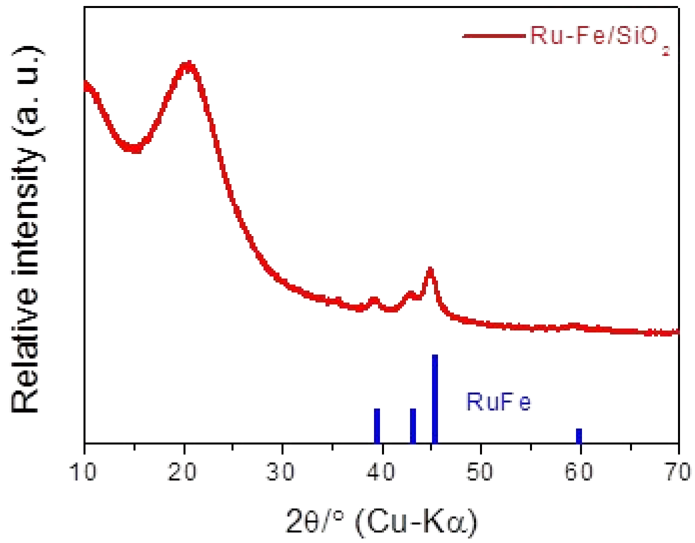

Fig. S3 XRD patterns of 5wt%Ru-5wt%Fe/SiO_2. The bottom blue columns represent the reference XRD patterns of RuFe (JCPDS No.65-6545).

We loaded 5wt%Ru-5wt%Fe onto the surface of amorphous SiO_2 under the same preparation condition as Ru-Fe/C12A7:e^-. As we expected, the RuFe alloy with a hexagonal close-packed phase (space group: P63/mmc, JCPDS No. 65-6545) is formed on the support.

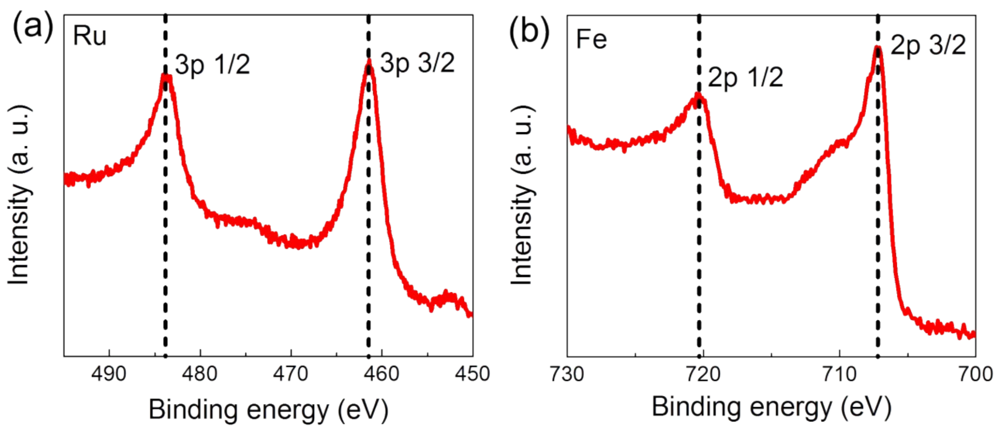

Fig. S4 XPS Ru 3p (a) and Fe 2p (b) spectra for Ru-Fe/C12A7:e⁻. The dotted lines indicate the Ru 3p and Fe 2p peaks derived from bulk Ru and Fe metal respectively.

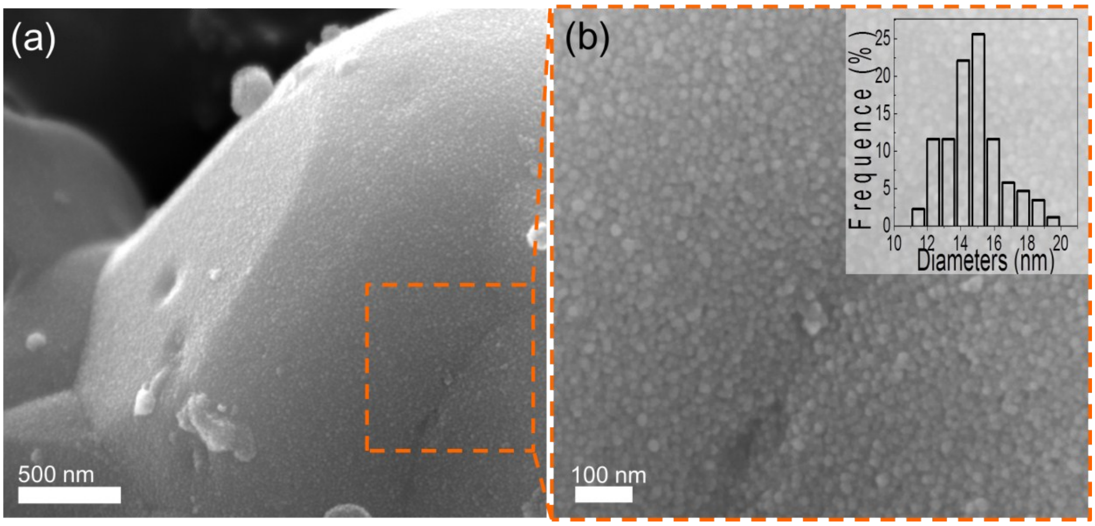

Fig. S5 SEM images of Ru-Fe/C12A7:e⁻ surface: overview (a) and enlargement of a homogeneous section (b) from (a). Inset (b): Metallic particle size distribution.

Table S1. Summary of reported Ru, Pt and other transition metal based heterogeneous catalysts for hydrogenation of cinnamaldehyde.

Catalyst	Solvent	B.E.T. [m^2g^{-1}]	Conv. [%]	Sel. [%]	Ref.
**Ru-Fe/C12A7:e^-**	**------**	**0.8**	**96.2**	**96.7**	**This work**
Ru/CNT	isopropanol	27	80	92	[S1]
Ru-Sn/SiO_2	isopropanol	297	99	59	[S2]
Pt-Ru/CNT1273	dioxane	246	78	94	[S3]
2% Pt 2% Ru/MWNT	isopropanol	196	79	93	[S4]
Ru/HSAG@mSiO_2	isopropanol	613	95	65	[S5]
Pt(2.41%)/LDH-EG	ethanol	87.8	93.6	78.8	[S6]
3.5 wt %Pt/G	isopropanol	57	92	88	[S7]
Pt(5.0%)/CNT-973	ethyl acetate	(EAS) 23^a	89.8	78.8	[S8]
0.3%Pt−0.1%Co/ZrO_2	ethanol	74.8	90.2	88.0	[S9]
0.5%Pt−0.17%Co/CNT	ethanol	110	92.4	93.6	[S10]
PtCo/0.5-CNx/TiO_2	ethanol	Unknown	97.1	79.3	[S11]
Pt(5%)/CeO_2–ZrO_2	isopropanol	94	95.8	93.4	[S12]
Pt(1.96%)/MA	water	Unknown	79.7	85.4	[S13]
PtNCs/UiO-66-NH_2	methanol	676	98.7	91.7	[S14]
Pt/RGO	isopropanol	276.1	89.6	69.6	[S15]
Pt-Co/Graphene	isopropanol	Unknown	94.6	89.5	[S16]
Pt/RGO	ethanol	343.2	97.8	85.3	[S17]
PtFeZn/C-UA	cyclohexane	800	96	86	[S18]
Pd–Sn/AC	isopropanol	Unknown	96	80	[S19]
Au/BP (Au; 1.1 mol%)	water	107	78	80	[S20]
Au–Ir/TiO_2	isopropanol	50	95	75	[S21]
AuNPs/TiO_2/reduced	isopropanol	60	61.6	49.3	[S22]
Ni/TiO_2	methanol	150	91.0	61.0	[S23]

^{a} Electrochemical active surface (EAS) areas of Pt nanoparticles by using CO stripping method.

Table S2. The catalytic performance for chemoselective hydrogenation of cinnamaldehyde.[a]

Entry	Catalyst	Ru : Fe [wt%]	H_2 [MPa]	Temp. [°C]	Time [h]	Conv. [%]	Sel. [%]
1	Ru-Fe/C12A7:e⁻	1:1	1.0	130	12	33.3	87.1
2	Ru-Fe/C12A7:e⁻	1:1	2.0	110	12	40.5	92.6
3	Ru-Fe/C12A7:e⁻	1:1	2.0	90	12	20.2	92.5
4	Ru-Fe/C12A7:e⁻	1:1	2.0	60	72	20.4	94.5
5	Ru-Fe/C12A7:e⁻	1:1	2.0	130	6	21.4	93.3
6[b]	Ru-Fe/C12A7:e⁻	1:1	2.0	130	12	----	----
7	Ru-Fe/C12A7:e⁻	2.5:2.5	2.0	130	12	96.9	85.6
8	Ru-Fe/C12A7:e⁻	5:5	2.0	130	12	98.6	80.7
9	Ru-Fe/C12A7:e⁻	2:1	2.0	130	12	98.1	76.6
10	Ru-Fe/C12A7:e⁻	10:1	2.0	130	12	98.0	----
11[c]	Ru-Fe/C12A7:e⁻	1:1	2.0	130	12	91.2	79.6
12[d]	Ru-Fe/C12A7:e⁻	1:1	2.0	130	12	93.4	91.3

[a] Typical conditions: 8 mmol cinnamaldehyde, 100 mg of catalyst;
[b] Substrate: 8mmol cinnamyl alcohol, 100 mg of catalyst;
[c] C12A7:e⁻ with $N_e = 1.2\times10^{20}$ cm^{-3};
[d] C12A7:e⁻ with $N_e = 1.1\times10^{21}$ cm^{-3}.

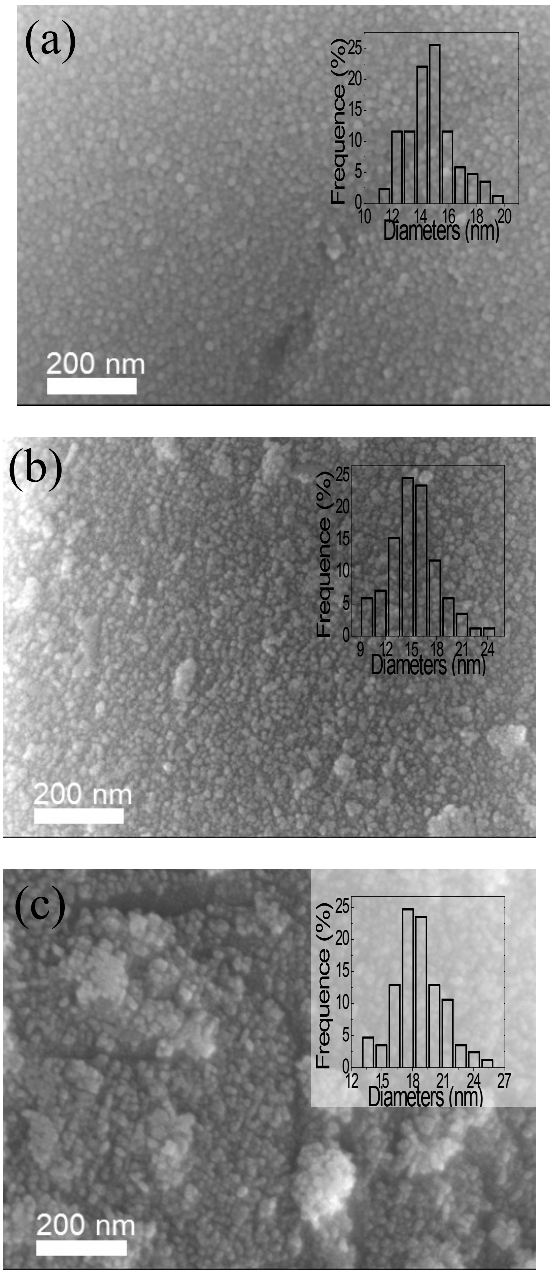

Fig. S6 SEM images of several bimetallic Ru-Fe/C12A7:e^{-} catalysts with different Ru/Fe weight ratios: (a) 1wt%Ru-1wt%Fe/C12A7:e^{-}, (b) 2.5wt%Ru-2.5wt%Fe/C12A7:e^{-} and (c) 5wt%Ru-5wt%Fe/C12A7:e^{-}. Inset: Metallic particle size distribution.

We appropriately tune of Ru-Fe loading amount from 2 wt% to 10 wt%, the mean size of the metal nanoparticles was increased from 15 to 20 nm with metal nanoparticles aggregated gradually. Accordingly, the selectivity to cinnamyl alcohol decreased a lot although the conversion slightly elevated (Table 1, entry 4; Table S2, entries 7−8). The larger nanoparticles aggregation would decrease the fraction of metal-support interface, reducing the selectivity to cinnamyl alcohol.

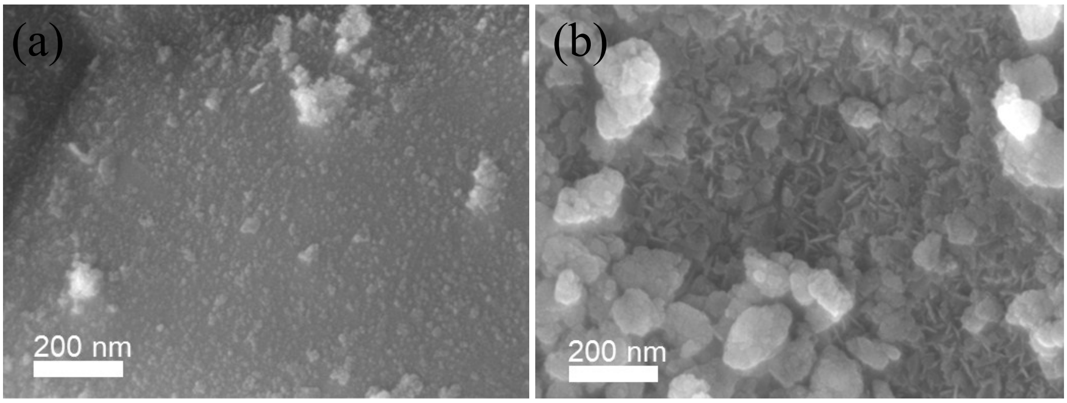

Fig. S7 SEM images of several bimetallic Ru-Fe/C12A7:e⁻ catalysts with different Ru/Fe weight ratios: (a) 2wt%Ru-1wt%Fe/C12A7:e⁻, (b) 10wt%Ru-1wt%Fe/C12A7:e⁻.

When Ru-rich catalyst were used (Table S2, entries 9−10), the selectivity decreased sharply, attributed to the superabundant of active metal Ru which can be clearly observed on the surface morphology of the catalyst.

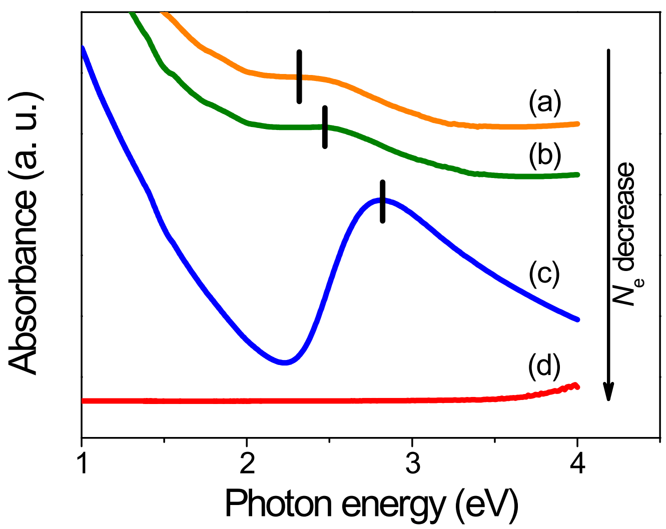

Fig. S8 The UV-vis absorption spectra (obtained by Kubelka-Munk tansformation of diffuse reflectance spectra) for the synthesized powders of C12A7:e^{-} or C12A7:O^{2-} with various electron concentrations (a) N_e =2.2×10^{21} cm^{-3}, (b) N_e =1.1×10^{21} cm^{-3}, (c) N_e =1.2×10^{20} cm^{-3}, (d) N_e =0 cm^{-3}.

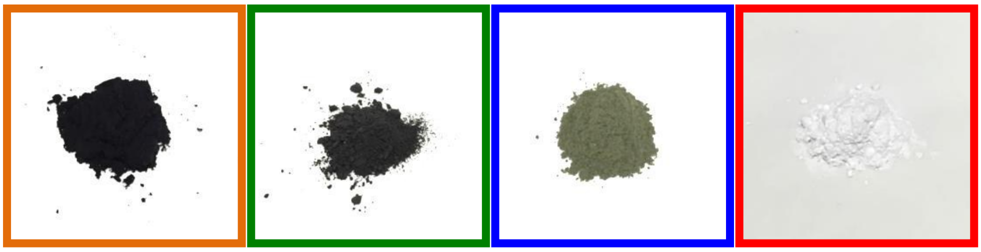

N_e =2.2×10^{21}cm^{-3} N_e =1.1×10^{21}cm^{-3} N_e =1.2×10^{20}cm^{-3} N_e =0 cm^{-3}

Fig. S9 The photographs of the synthesized powders of C12A7:e^- or C12A7:O^{2-} with various electron concentrations.

The sample color was changed from white by green to black when N_e increased from 0 to 2.2×10^{21} cm^{-3}, which is good agreement with the colors of C12A7 single crystals treated with Ca metal in a vacuum. There is no adsorption peak of C12A7:O^{2-} sample in the visible region, and the corresponding 3.5 eV absorption edge is attributed to the excitation between the energy level of encaged O^{2-} ions and the cage conduction band (CCB). By contrast, C12A7:e^- samples give broad absorption bands at 2−3.5 eV and below 2 eV. The former absorption is due to an intracage s-to-p transition of electrons trapped in the cages, and the latter is attributed to an intercage s-to-s transition as charge transfer from an electron-trapped cage to a vacant neighboring cage. The absorption band below 2 eV is broadened, and the band tail extends to larger energies, which is affected by the disorder of the extra framework species. It was therefore confirmed that O^{2-} ions accommodated in the cages of C12A7:O^{2-} are replaced by electrons by heat treatment with Ca metal in a vacuum.

Table S3. Electron Concentration (N_e) of C12A7:e^- or C12A7:O^{2-} samples.

N_e^a (10^{21}cm^{-3})	N_e^b (10^{21}cm^{-3})
2.2	2.1
1.1	1.5
0.12	0.10
0	0

$^a N_e$ was determined by iodometric titration method. $^b N_e$ was determined by the relation of $N_e = [-(E_{sp} - E_{sp}°)/0.199]^{0.782}$.

N_e can be estimated from E_{sp} using the experimentally obtained relation of $N_e = [-(E_{sp} - E_{sp}°)/0.199]^{0.782}$, where the low-Ne limit $E_{sp}°$ = 2.83 eV at $N_e \approx 1 \times 10^{18}$ cm^{-3}. The obtained values correspond well with those determined by the iodometric titration method.

Table S4 Chemoselective hydrogenation of benzalacetone using Ru-Fe /C12A7:e⁻.[a]

Entry	Substrate	Product	Conv. [%]	Sel. [%]
1	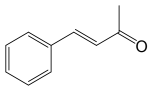	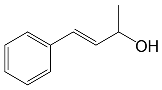	42.2	15.1

[a] Typical conditions: 8 mmol substrate, 100 mg catalyst, H_2 (2.0 MPa), 90 °C, 24 h.

In the case of chemoselective reduction of α,β-unsaturated ketone, we tried the hydrogenation of benzalacetone under solvent free system and the conversion and selectivity was quite poor compared with cinnamaldehyde (entry 1). Probably, the steric hindrance by the methyl group in α,β-unsaturated ketone impeded adsorption of the C=O bonds to the Ru-Fe active sites significantly.

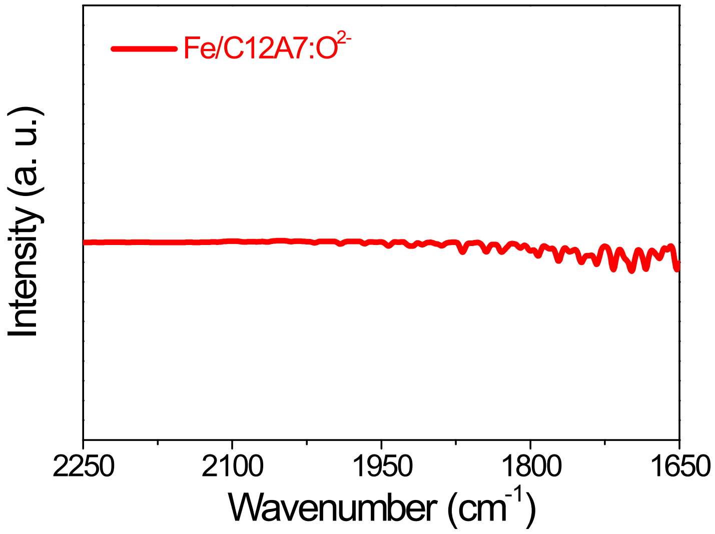

Fig. S10 Difference DRIFTS spectrum of CO molecules adsorption on Fe/C12A7:O^{2-} at −170 ° C under 5 KPa of CO.

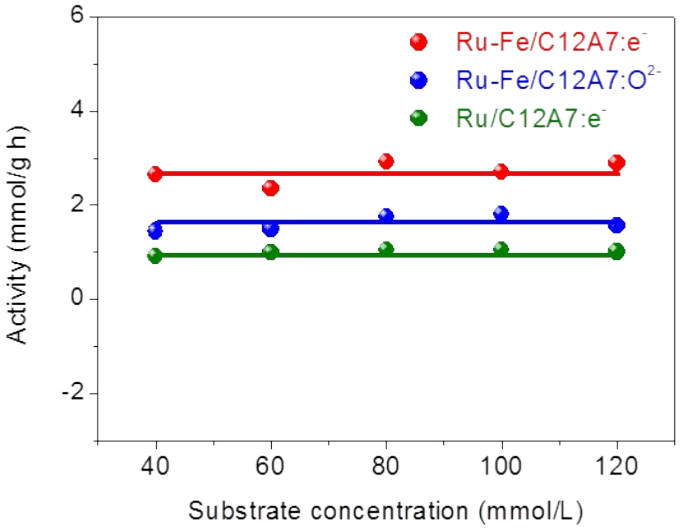

Fig. S11 Effect of substrate concentration on the rates of hydrogenation of cinnamaldehyde (Catalyst: 5 mg; THF: 5 mL; 2MPa H_2, 130 °C; 12h).

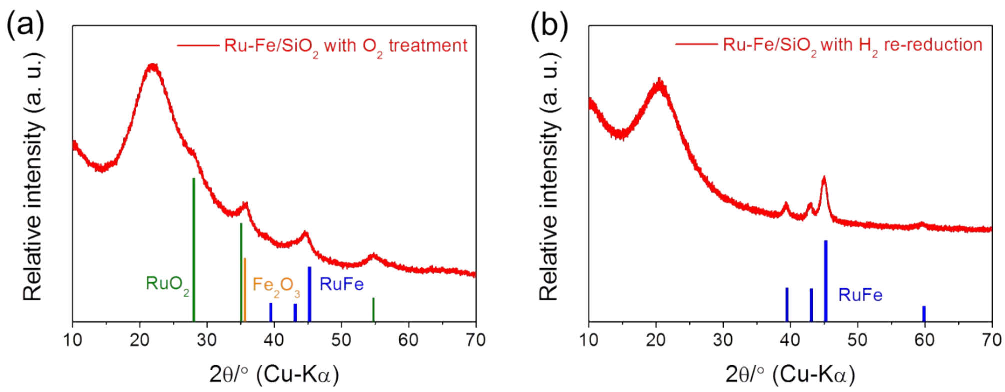

Fig. S12 XRD patterns of (a) 5wt%Ru-5wt%Fe/SiO_2 pretreated in O_2 (30 mL/min) at 623 K for 30 min; 5wt%Ru-5wt%Fe/SiO_2 re-reduction in H_2/Ar (30 mL/min) at 623 K for 30 min. The bottom blue, green and orange columns represent the standard JCPDS diffraction pattern of RuFe (JCPDS No.65-6545), RuO_2 (JCPDS No.40-1290), and Fe_2O_3 (JCPDS No.39-1346), respectively.

We measured the XRD pattern of 5wt%Ru-5wt%Fe/SiO_2 with oxidation treatment, which is the same as the pretreatment of H_2-TPR (O_2, 623 K, 30 mins). Part of the hexagonal RuFe alloy phase remained and RuO_2 and Fe_2O_3 phases were also observed in the XRD pattern (Fig. R1a). Although phase separation between Ru oxide and Fe oxide occurred during the oxidation pretreatment, the re-reduction process brought about the RuFe alloy formation again (Fig. R1b). Therefore, we think that RuO_2 and Fe_2O_3 are present in close proximity to each other on the surface of the RuFe alloy after the oxidation treatment. As a result, H_2-TPR peaks for Ru-Fe catalysts are observed in the intermediate temperature region between that of Ru and Fe catalysts (Fig. 3a).

References

[S1] J. M. Planeix, N. Coustel, B. Coq, V. Brotons, P. S. Kumbhar, R. Dutartre, P. Geneste, P. Bernier and P. M. Ajayan, *J. Am. Chem. Soc.*, 1994, **116**, 7935–7936.

[S2] J. Hájek and D. Y. Murzin, *Ind. Eng. Chem. Res.*, 2004, **43**, 2030–2038.

[S3] J. Teddya, A. Falquib, A. Corriasc, D. Cartac, P. Lecanted, I. Gerbere and P. Serpa, *J. Catal.*, 2011, **278**, 59–70.

[S4] H. Vu, F. Goncalvesa, R. Philippea, E. Lamourouxa, M. Corriasa, Y. Kihnb, D. Pleec, P. Kalcka and P. Serp, *J. Catal.*, 2006, **240**, 18–22.

[S5] H. Shen, H. Tang, H. Yan, W. Han, Y. Li and J. Ni, *RSC Adv.*, 2014, **4**, 30180–30185.

[S6] Y. Wang, W. He, L. Wang, J. Yang, X. Xiang, B. Zhang and F. Li, *Chem. Asian J.*, 2015, **10**, 1561–1570.

[S7] X. Ji, X. Niu, B. Li, Q. Han, F. Yuan, F. Zaera, Y. Zhu and H. Fu, *ChemCatChem*, 2014, **6**, 3246 –3253.

[S8] Z. Guo, Y. Chen, L. Li, X. Wang, G. L. Haller and Y. Yang, *J. Catal.*, 2010, **276**, 314–326.

[S9] X. Han, R. Zhou, B. Yue and X. Zheng, *Catal. Lett.*, 2006, **109**, 157–161.

[S10] Y. Li, P. Zhu and R. Zhou, *Appl. Surf. Sci.*, 2008, **254**, 2609–2614.

[S11] Z. Tian, Q. Li, Y. Li and S. Ai, *Catal. Commun.*, 2015, **61**, 97–101.

[S12] S. Bhogeswararao and D. Srinivas, *J. Catal.*, 2012, **285**, 31–40.

[S13] X. Xiang, W. He, L. Xie and F. Li, *Catal. Sci. Technol.*, 2013, **3**, 2819–2827.

[S14] Z. Guo, C. Xiao, R. V. Maligal-Ganesh, L. Zhou, T. W. Goh, X. Li, D. Tesfagaber, A. Thiel and W. Huang, *ACS Catal.*, 2014, **4**, 1340−1348.

[S15] Z. Sun , Z. Rong, Y. Wang, Y. Xia, W. Du and Y. Wang, *RSC Adv.*, 2014, **4**, 1874–1878.

[S16] Z. Rong, Z. Sun, Y. Wang, J. Lv and Y. Wang, *Catal. Lett.*, 2014, **144**, 980–986.

[S17] J. Shi, R. Nie, P. Chen and Z. Hou, *Catal. Commun.*, 2013, **41**, 101–105.

[S18] N. Mahata, F. Gonçalves, M. Fernando, R. Pereira and J. Figueiredo, *Appl. Catal. A Gen.*, 2008, **339**, 159–168.

[S19] J. Zhao, X. Xu, X. Li and J. Wang, *Catal. Commun.*, 2014, **43**, 102–106.

[S20] M. M. Wang, L. He, Y. M. Liu, Y. Cao, H. Y. He and K. N. Fan, *Green Chem.*, 2011, **13**, 602–607.

[S21] J. Zhao, J. Ni, J. Xu, J. Xu, J. Cen and X. Li, *Catal. Commun.*, 2014, **54**, 72–76.

[S22] Y. Liu, Y. Luo and Z. Wei, *Chem. Asian J.*, 2013, **25**, 8617–8620.

[S23] M. G. Prakash, R. Mahalakshmy, K. R. Krishnamurthy and B. Viswanathan, *Catal. Sci. Technol.*, 2015, **5**, 3313–3321.
